# Lived experiences of student nurses caring for intellectually disabled people in a public psychiatric institution

**DOI:** 10.4102/curationis.v39i1.1601

**Published:** 2016-06-30

**Authors:** Annie Temane, Lizzie Simelane, Marie Poggenpoel, CPH Myburgh

**Affiliations:** 1 Department of Nursing, University of Johannesburg, South Africa; 2 Department of Educational Psychology, University of Johannesburg, South Africa

## Abstract

**Background:**

Caring for intellectually disabled people can be demanding for student nurses who are novices in the nursing profession. To ensure that quality nursing care is provided, student nurses should have an understanding of and a positive attitude towards intellectually disabled people. Nursing intellectually disabled people can be a challenge for the student nurses. Therefore, student nurses need to be able to deal with challenges of caring for intellectually disabled people.

**Objective:**

This article aims to explore and describe experiences of student nurses caring for intellectually disabled people in a public psychiatric institution.

**Design and method:**

A qualitative, exploratory, descriptive and contextual research design was used. Data were collected through individual in-depth phenomenological interviews, naïve sketches and field notes. Thematic analysis was utilised to analyse the collected data. Results were contextualised within the literature and measures to ensure trustworthiness were adhered to. Ethical principals were also applied throughout the research process.

**Results:**

Five themes emerged from the data. Student nurses experienced a profoundly unsettling impact on their whole being when caring for intellectually disabled people; they developed a sense of compassion and a new way of looking at life, and experienced a need for certain physical, mental and spiritual needs to be met.

**Conclusion:**

From the results, it is evident that student nurses were challenged in caring for intellectually disabled people. However, they developed a sense of awareness that intellectually disabled people have a need to be cared for like any other person.

## Introduction

Most institutionalised people’s intellectual disabilities are severe and profound, and characterised by physical disabilities, aggression, low frustration tolerance and self-injurious behaviours (Sadock, Sadock & Ruiz 2015:1135). These challenging behaviours may directly affect the mental health of those who care for them. Caring for intellectually disabled people can be demanding for student nurses who are novices in the profession. Kersey-Matusaik (2012:84) indicates that student nurses need to be prepared to care for patients with long-term challenges such as intellectual disabilities. Intellectually disabled people require a caring relationship that enables an enhanced awareness of life and health experiences. This relationship facilitates health and healing processes as it involves trustworthiness and the genuine needs of patients.

Nursing is a challenging and complex job. The work is exhilarating, rewarding and exhausting and can also be heart-breaking as nurses work with people who are in the depths of suffering (Davey *et al*. 2016:496). A study conducted by Bakker and Demeroutti (2014:3) revealed that some job characteristics such as caring for intellectually disabled people can have a profound impact on the carer’s well-being and may cause work-related stress. Work-related stress is the result of a conflict between the role needs of an individual and demands of the work place. Stress has been described in a variety of ways to reflect environmental demands, the person’s response to the demands or factors influencing the person and coping responses in relation to the cognitive appraisal (Najimi, Goudarzi & Sharifirad 2012:301). The results of stress in nursing are a decrease in morale and performance and hence may affect the quality of patient care (Lewis & Stenfert-Kroese 2009:356).

Student nurses need to be prepared with regard to caring for patients with long-term illnesses such as intellectual disabilities. These patients require a caring relationship that enables enhanced conscious awareness of life and experiences. The caring relationship facilitates health and the healing process as it involves the authentic and genuine needs of patients (Berg & Danielson 2007:500). Janse van Rensburg (2009:2) states that student nurses experience emotional discomfort while working with intellectually disabled people. She also claims that the environment is a challenging context that makes adjustment difficult for student nurses.

Stigmatisation and exclusion of the intellectually disabled people take place because people respond to the fact that those who are intellectually disabled are different. The difference lies in their challenges which include suffering and dependency on others (Ditchman *et al*. 2013:206). Lack of knowledge and understanding may influence student nurses in such a way that it leads to negative attitudes towards intellectually disabled people (Awoyera 2011:8).

### Problem statement

Mental health nursing is part of the basic education of student nurses to prepare them to work as comprehensive health professionals (Uys & Middleton 2014:11). Student nurses form part of the working force to care for intellectually disabled people in order to gain clinical experience in regard to their clinically expected outcomes to meet the requirements of registration with the South African Nursing Council (SANC), according regulation 425, leading to registration as a general, community, psychiatric nurse and midwife.

Intellectual disability is a lifelong condition and the management thereof must constantly be adapted to satisfy the needs of the individual affected. To ensure that optimal nursing care is received, student nurses must have both an understanding of and knowledgeable attitude towards intellectually disabled people. The researcher observed during contact sessions with student nurses that they felt challenged in caring for intellectually disabled people. Some distance themselves from the clinical context, while others express a fear of caring for intellectually disabled people.

There is little known about how student nurses experience caring for intellectually disabled people. The research question that arose was: “What are the lived experiences of student nurses caring for intellectually disabled people?”

## Aim of the study

This article aims to explore and describe lived experiences of student nurses when caring for intellectually disabled people in a public psychiatric institution.

## Definition of key concepts

*Experience*: Experience is knowledge and skills gained through time spent doing a job or activity (Merriam-Webster Online Dictionary 2016:1). In this study, the term refers to practical contact with an observation of facts, events, knowledge or skills gained over time.

*Student nurse*: A ‘student nurse’ is a learner registered for a 4-year course leading to registration as a comprehensive, general, psychiatry and community, and midwifery, according to the SANC, Regulation R425 of the 22 February 2005 as amended, and function under the supervision of a registered nurse. In this study, a ‘student nurse’ refers to a fourth-year student nurse who has cared for intellectually disabled people in a public psychiatric institution.

*Caring*: ‘Caring’ is beneficence or helping (Jooste 2010:6). In this study, it relates to the reduction of suffering and the fostering of growth and development of a human being in suffering.

*Intellectually disabled people*: It refers to people with intellectual disabilities. Intellectual disabilities include a range of intellectual functioning disabilities extending from partial self-maintenance under close supervision, together with limited self-protection skills in a controlled environment through limited self-care and requiring constant aid and supervision, to severely restricted sensory and motor functioning and requiring nursing care.

*Public psychiatric institution*: It refers to a health establishment, a centre, organisation, a unit or part thereof, which is devoted primarily to the diagnosis, treatment, care, rehabilitation or detention of intellectually disabled people (*Mental Health Care Act* no 17 of 2002:7). In this study, it means a hospital specialising in the treatment of serious mental disorders.

*Research significance*: There is a need to describe the lived experiences of student nurses caring for intellectually disabled people in a public psychiatric institution.

## Research design

A post-modern constructivist philosophy of science (Creswell 2013:42) was adhered to in this research. This philosophy relates to how individuals construct meaning and their interpretation as they engage with their own world (Creswell 2014:9) A qualitative exploratory descriptive and contextual research design was utilised in this study (Burns, Grove & Grey 2013:359; Creswell 2013:175; Edmonds & Kennedy 2013:112 & 130).

## Research method

A hermeneutical phenomenology approach was followed in this research in order to explore the meaning of the lived experiences of participants in a study (Creswell 2013:79).

A description will be given below about the research setting, population and sample, data collection, data analysis, measures for trustworthiness and ethical measures.

### Research setting

The study took place at a public psychiatric institution which caters only for the needs of intellectually disabled people. Student nurses are placed at this institution to care for and meet the needs of the intellectually disabled people to meet their clinically expected outcomes.

### Population and sampling

The population of this study was student nurses who were placed at a public psychiatric institution for clinical learning. The total population of the student nurses in the public psychiatric institution was 30. Purposive sampling is based on the judgement of the researcher regarding participants that are representative of the study (Brink 2009:133) and was used to select student nurses who have experienced caring for intellectually disabled people in a public psychiatric institution. The sample size of 12 nursing students was utilised in this study. The inclusion criteria were considered on the basis that student nurses had cared for intellectually disabled people in a public psychiatric institution, were willing to share their experiences and were in their fourth year of the study.

### Data collection

Individual in-depth interviews (Rubin & Rubin 2012:25) were used to collect data about the lived experiences of student nurses caring for intellectually disabled people in a public psychiatric institution. Ten student nurses were interviewed and two wrote naïve sketches (Giorgi 2009:10). One central question was asked: “*How was it for you to be working at this institution?”* The interviews were audio recorded and field notes (Botma *et al*. 2010:49–50) were taken. Data were collected until data saturation (Burns *et al*. 2013:361) was reached as evidenced in repeating themes.

### Data analysis

Audiotapes were listened to and transcribed verbatim. The transcriptions, naïve sketches and field notes were analysed by means of thematic analysis according to steps proposed by Braun and Clarke (2007:80). A clean set of transcriptions, naïve sketches and field notes was sent to an independent coder for analysis. After a consensus discussion between the researcher and independent coder, relevant themes and categories were established about the lived experience of student nurses caring for intellectually disabled people. Thereafter, the results were supported by a literature control.

### Measures for trustworthiness

The method of establishing trustworthiness was adapted from that of Lincoln and Guba’s model (De Vos *et al*. 2011:419). Credibility, dependability, transferability and confirmability strategies were employed (see Table 1 for the strategies applied).

**TABLE 1:** Trustworthiness strategies applied in the study.

Strategy	Criteria	Application
Credibility	Prolonged engagement	Building trust by honouring anonymity
		Data saturation through data collection of individual, semi- structured interviews
	Triangulation	Multiple methods of data collection (individual in-depth interviews, naïve sketches and field notes)
		Literature control
		Use of independent coder
	Peer evaluation	Three supervisors with extensive qualitative research experience
		Study was presented at a national research forum
	Member checking	Informal member checking was done during interviews by clarifying, summarising and discussions with participants
		Discussions with supervisors
Transferability	Dense description	Results are described in-depth with direct quotations from interviews
		Results are re-contextualised in literature
	Nominated sample	Detailed description of the demographic information of participants was provided
		Sampling method utilised was described
Dependability	Audit trail	Transcripts materials and field notes will be kept as audit trail
	Dense descriptions of research methods	Detailed description of the data collection and data analysis was outlined.
	Code–recode procedure	Consensus meeting and discussion was held with the independent coder after data analysis
	Triangulation	As discussed
	Peer evaluation	As discussed
Confirmability	Audit trail	Transcripts and naïve sketches will be kept for audit inquiry
	Triangulation	As discussed
	Peer examination	As discussed

*Source*: De Vos *et al*. (2011:419)

### Ethical considerations

Student nurses were informed about the aims of the study and the option to participate and the right to withdraw at any time without being penalised. Written consent was obtained from the student nurses to participate in the study and to be audiotaped during the interviews. Student nurses were assured of their anonymity and confidentiality.

Approval and permission to conduct the study was obtained from the Faculty of Health Sciences Research Ethics Committee of the University of Johannesburg (Ref number AEC 20-01-2013), the University of the Witwatersrand, Human Research Ethics Committee (Ref number M130522) and the Gauteng Provincial Protocol Review Committee (Ref number P090813).

## Results

Five main themes concerning the experiences of student nurses when caring for intellectually disabled people in a public psychiatric institution emerged from the data analysis. Themes and categories are summarised in Table 2. Findings are discussed together with the literature control.

**TABLE 2:** Themes and categories.

Themes	Categories
Experience of a profound unsettling impact on their wholistic being when caring for intellectually disabled people	Student nurses experience that for intellectually disabled people has an effect on their body mind and spirit
Experience of a sense of compassion and a new way of looking at life	Student nurses experience that they developed awareness for caring for intellectually disabled people
Experience of a need to cope while caring for intellectually disabled people	Student nurses experience educational, emotional and spiritual needs when caring for intellectually disabled people

*Source*: Simelane (2015:37)

### Theme 1: Experience of a profoundly unsettling impact on their holistic being when caring for intellectually disabled people

From the data collected, student nurses gave a detailed description of the profoundly unsettling impact on their holistic being. According to the Theory for Health and Promotion in Nursing, an individual is viewed as being whole, that is, he and/or she embodies the dimensions of body, mind and spirit (University of Johannesburg 2010:4). Thus student nurses reported that their body, mind and spiritual well-being were affected.

Student nurses experienced that caring for intellectually disabled people had unintended consequences on their body, mind and spirit. They reported experiencing lack of appetite when caring for intellectually disabled people. As one student nurse reported:

‘It was difficult emotionally and physically because we have to take care of them whether we like it or not. They defecate on the floor and they eat it and afterwards they want to touch you with those hands. I could not eat every time I think about them, that was a terrible situation.’ (Participant 9)

Another stated that:

‘Thinking of food after being in such an environment was difficult. I lost weight in one week.’ (Participant 10)

The student nurses’ ability to think and reasoning was affected in relation to their own view on parenthood and they realised the needs of the intellectually disabled people. Caring for intellectually disabled people created doubts for student nurses regarding parenthood and whether they should or should not become parents. This is supported by the following quotes:

‘Seeing all those kids, it makes you not to want to have a kid of your own.’ (Participant 9)

And:

‘It was unsettling in a way because you thinking that what if my child becomes like that.’ (Participant 8)

In this study, the spirit is related to the struggle with ethical issues arising when caring for intellectually disabled people, inner conflict and the struggle to see the purpose of intellectually disabled people. Student nurses experienced inner conflict when caring for intellectually disabled people, causing them to feel very overwhelmed, depressed, scared, anxious, sad and heartbroken. They lost their drive and motivation to cope with the situation. One student nurse wrote this narrative:

‘Truly speaking, I do not have that strong heart, I just broke down and cried in front of those people. …To think that they will just live the rest of their lives not knowing how being cared for and being loved feels like.’ (Participant 2)

One student nurse commented:

‘The only challenge for me was seeing those people, they broke my heart.’ (Participant 1)

Student nurses struggled to see the purpose of the intellectually disabled people’s lives and could not make sense of it. They asked themselves why intellectually disabled people are in the world:

‘I really do not understand why such people live. And to think that they live longer with all those deformities and all the suffering, even if they are not aware of the sufferings but other people do.’ (Participant 4)

And:

‘I personally think that this people are just here to increase the country statistics, eat and sleep with no purpose in life.’ (Participant 1)

### Theme 2: Compounding factors that affected caring for intellectually disabled people

Student nurses experienced that caring for intellectually disabled people was challenging in regard to cognitive disabilities and physical deformities that are demonstrated by limited motor and speech development, unhygienic environment and the way food was served to intellectually disabled people.

Intellectually disabled people react differently towards internal and external environments and their language and speech are affected. Their limited speech abilities led them not to be able to express their wants and needs so they made sounds that came out as a scream, a laugh, or noises. These sounds and noises had a negative effect on student nurses. One of the students said:

‘The sounds that they were making…, so these sounds stay in your head even when you are at home, when you are in a quiet place you still hear these sounds.’ (Participant 2)

Another participant commented:

‘It was traumatic to hear the sounds that they were making, it was unrealistic.’ (Participant 8)

Student nurses experienced varying levels of discomfort and anxiety when caring for intellectually disabled people who were different from them in terms of physical appearance. One student reported:

‘It was for the first time that I saw so many intellectually disabled people, all those with hydrocephalus, some have deformed limbs. Seeing these patients I started having diarrhoea. Their limbs were so stiff.’ (Participant 8)

Another student nurse said:

‘They were very, very crippled, not merely severe but profoundly crippled and they could not do anything for themselves.’ (Participant 9)

Student nurses experienced intense emotional discomfort and uncertainty with regard to physically handling intellectually disabled people. The discomfort was attributed to perceived lack of skill in handling these people.

‘Some of them the limbs are so stiff…. hands that are immobile, you have to pull very hard when dressing them, it hurts them, you become frustrated because if you do not dress them they will get cold. You really do not know exactly what to do next.’ (Participant 6)

And the other student nurse said:

‘Most of the children had severe contractures. I did not know how to approach such a situation. We were never given a course to say that if somebody is like this and you need to attend to him this way.’ (Participant 8)

Furthermore, they reported that the environment where in the intellectually disabled where care for was unhygienic. Due to so many intellectually disabled people being institutionalised, public psychiatric institutions are overcrowded. Human and material shortages make the situation worse. The wards are unclean and unhygienic, with some rooms and surrounding areas foul-smelling and covered with faeces and urine. Student nurses were negatively affected by the smells that they encountered when caring for intellectually disabled people:

‘The environment was not healthy and by not healthy I mean the air you breaths smells. It is not acceptable to nurse people in such a smelly environment.’ (Participant 5)

Another said:

‘The place was not suitable for nursing people, it was unhygienic, there were faeces all over the place. The place stinks.’ (Participant 10)

Another compounding factor that student nurses experienced was the way food was served to intellectually disabled people was inhumane. Student nurses elaborated:

‘Feeding time was very difficult. The food was horrible, yoo!!! The food was horrible (student nurse covering her face with her hands, bending over the table), okay, the food is not horrible per se but the way they prepare it is. They mix it. In soft porridge they will put bread, they put the polony in there, they pour in the tea, mix it up to form a brownish slop and give it to them. It make sense that feeding would take a long time if you give one item at a time but honestly, it was food that you would not give a normal person to eat. It felt like we were treating them like pigs.’ (Participant 8)

Another student added:

‘The food itself was something else. It was a mixture of all kinds of foods, bread, tea, cereal and porridge mixed in a big bowl. Probably in there, they are not regarded as human beings because of the way they are treated.’ (Participant 10)

### Theme 3: Experienced dehumanising care of intellectually disabled people

Student nurses experience that intellectually disabled people are treated unfairly with regard to quality of nursing care and are being abused and discriminated against. One of the student nurses said:

‘In terms of the caring itself, you know, at times the patients are beaten. Here at the facility, there are those boys who their mental state is questionable but for them to be beaten by some staff members to calm them and behave in a proper manner … even though they are disabled, they need to be treated fairly.’ (Participant 1)

Student nurses experienced an awareness of the injustices towards intellectually disabled people and they were of the opinion that these people are treated unfairly, not respected, discriminated against and stigmatised. One student said:

‘The place is on the outskirts and makes you feel that the facility is for the unwanted.’ (Participant 3)

Another student nurse reported:

‘To me that place is a prison, it is as if they are not accepted in the society and they just do not fit in, plus that facility is very far and isolated from the community. The facility should not be so isolated; intellectually disabled people also need to interact with the community.’ (Narrative 2)

### Theme 4: Experience a sense of compassion and a way of looking at life

Student nurses encountered many problems at a public psychiatric institution but were aware of what was needed. They developed a sense of compassion and gratitude. Gilbert (2005:1) states that compassion involves being open to the suffering of self and others in a non-judgmental way. It also involves a desire to relieve suffering, cognitions related to understanding the cause of suffering, and behaviours. The person giving compassion grows and in some ways become in tune with the feelings and needs of others (Gilbert 2005:44).

Although student nurses were initially overwhelmed while caring for intellectually disabled people, they also experienced that they developed a deep level of empathy towards intellectually disabled people, as the following quotes illustrate:

‘Yes they are demanding, yes okay they are demanding but at the end of the day they are human beings.’ (Participant 3)

And:

‘It broke my heart to think that there is nothing that can be done to help these people.’ (Participant 2)

During the period of clinical placements, student nurses realised that intellectually disabled people also have needs. This is seen in the following quotes:

‘They need extra, extra care, I mean helping with their diapers, helping them with feeding, giving them that loving, that special attention.’ (Participant 8)

Then:

‘I think they need more people, I think they need more volunteers to assist with play time, feeding because they all need that care. Each and every one of them is different; they all need that special care.’ (Participant 6)

Student nurses not only developed empathy for intellectually disabled people but also for the nursing personnel working at the public psychiatric institution. Student nurses placed themselves in the working environment and conditions that the permanent personnel are facing every day.

‘The staff was minimal and had a lot of work to do, they are really overworked.’

And:

‘Staff there are always tired and burn-out, it take a lot of effort to feed, to take care for these people. If they could really increase the staff because if people a tired, it means they are not giving the care that they should, what the children need.’ (Participant 9)

The intellectually disabled people, irrespective of their mental and physical disabilities, were accepted as human beings by the student nurses.

Student nurses experienced an awareness that families and communities need to take responsibility to care for intellectually disabled people and need empowerment to become involved. This is supported by the following quotes:

‘Families need to be involved, play a major role in caring for these children.’ (Participant 4)

‘The children need help from the community as well. Volunteers could really help with the hard work and families could also help out by taking the users for weekend pass outs.’ (Narrative 1)

And:

‘Institutionalisation is not fair for the patients, it is better if a patient is taken care of by family who has love for the child… in the ward, a patient does not get his personal space.’ (Participant 9)

Student nurses also experienced an awareness of personal abilities, which was worrying, overwhelming and frustrating, but nevertheless made them grow professionally and personally:

‘Now I can be able to counsel people that have chronic conditions, I will tell them about the children at the facility who has all the deformities and there is no medication to reserve their conditions whereas chronic conditions have treatment to control as long as you are compliant.’ (Participant 8)

Student nurses experienced a sense of gratitude when caring for intellectually disabled people. They learnt to appreciate their own level of health after seeing the conditions and deformities that the intellectually disabled people have:

‘We take for granted the gift of good health, ability to think proper and make decision.’ (Participant 1)

‘Working at the facility was an eye-opener in a sense that we take life for granted, we are healthy and are able to do things by ourselves, we are able to think.’ (Participant 2)

And:

‘In life we must appreciate what we have.’ (Participant 9)

Student nurses experienced an awareness of their own opportunities in life and were able to embrace these. They appreciated the way in which the uniqueness of intellectually disabled people challenged and stretched their capacity for compassion and wisdom, acceptance and understanding.

‘For me, that facility was an eye-opening experience, to see another world of life.’ (Participant 1)

And:

‘We should appreciate that we can wake up in the morning and do things for ourselves.’ (Participant 9)

### Theme 5: Experience a need to be supported whilst caring for intellectually disabled people

Student nurses reported a need to be supported by their tutors when caring for intellectually disabled people. Limited physical presence of tutors in clinical placement had an effect on their emotional and psychosocial needs. Student nurses reported that they need to be guided, supported and mentored by their tutors in clinical placement.

They were of the opinion that their educational needs were neglected when they were at the public psychiatric institution. This is emphasised by the following comments:

‘We were sent there alone. A tutor came once and he did not come towards … he asked us if we had settled and left after few hours and that was it, so it did not help much.” (Participant 3)

And:

‘Our tutors need to be always with us to guide and support but our tutors came once to visit us but did not stay long and he did not come to the wards he ended in one of the offices, so we were left on our own to experience all these.’ (Participant 9)

Student nurses experienced a need for practical guidance during training sessions of how to physically handle the intellectually disabled people. They were of opinion that they lack knowledge and skills to physically handle the intellectually disabled people. They were aware of their shortcomings but did not have a platform to express their feelings because of inadequate accompaniment. This is supported by:

‘They did not teach us some of their procedures… I did not learn anything; it was so bad for me because they deal with people that are not like me. We did not know how to approach such situation and were never given a course to say that somebody is like this and you need to attend to him like this.’ (Participant 10)

Student nurses experienced a need for guidance, support and effective role modelling of the public psychiatric institution’s personnel. They reported that it was challenging to care for intellectually disabled people and nursing personnel were not helping in giving them guidance and support. The institution’s personnel were not good role models. One student nurse said:

‘First day, the sister was not welcoming … Then every day we had to copy what they were doing and sometimes they were not treating those children right.’ (Participant 2)

And:

‘The facility was the worst educational institution I have ever experienced during my four year of study, from management, no professionalism displayed by called HoD.’ (Participant 5)

Student nurses experience emotional needs because they were working in a rapidly changing and stressful environment. They needed more emotional support to develop compassionate care. Student nurses indicated that they need to be prepared before working in a public psychiatric institution. This is supported by these comments:

‘I think that we needed to have been given a thorough psychological preparation that where we were going is not a place like a normal place.’ (Participant 10)

And:

‘Going to that place is a trauma that I will not forget and I think that it will better that student nurses and staff to go for counselling before and after the experience.’ (Participant 9)

Student nurses experienced a need for support through group discussions while working in the public psychiatric institution to ventilate. It was evident from the interviews that student nurses lacked support. It was also apparent that they need support systems of various types in their clinical setting. One student nurse said:

‘We were left on our own”. (Participant 1)

Another said:

‘The matron and sisters in the wards were not friendly, and they were not willing to help.’ (Participant 2)

Student nurses experienced a need for reflective group discussion after working in the public psychiatric institution. Structured moments for reflection can be helpful in the prevention of disproportional stress levels, low morals and burnouts experienced by students. The following comments by the student nurses indicated a need for reflective group discussions:

‘We felt so lost and started to feel that the one week we stayed was so long.” (Participant 4)

And:

‘It was so hard you become burn-out, it led you to become snappy and short tempered.’ (Participant 8)

Student nurses had spiritual needs when caring for intellectually disabled people. Spirituality helps a person appreciate oneself and the space in which one lives, breathes and works. Spiritual beliefs guide how you feel about issues ranging from living and dying, to understand the meaning of life and faith. Student nurses were challenged as to why God allows the sufferings and meaningless the intellectually disabled people. They experienced a need for guidance by a spiritual director. This is supported by:

‘I even pray to God and wondered why He keep such people in this world to suffer.’ (Participant 8)

‘We should not have kids like that in life’ (Participant 9),

And

‘These people are suffering for no reason.’ (Participant 1)

## Discussion of results

From the data collected, student nurses gave a detailed description of the profoundly unsettling impact caring for intellectually disabled people have on their holistic being. According to Dodge *et al*. (2012:223) being holistic recognises the totality of the human being, the interconnectedness of body, mind, emotions, spirit, social and cultural relationships, context and environment. Student nurses experienced that caring for intellectually disabled people had unintended consequences on their body, mind and spirit.

They experienced lack of appetite when caring for intellectually disabled people. Reduced appetite in student nurses is sometimes caused by situational stressors. Situational stress is associated with psychosomatic complaints, one being a lack of appetite and loss of weight. It occurs when an individual is unable to adjust or cope with a particular stressor, in this instance, unhygienic smells in the wards acted as stressors to the student nurses. The stress usually resolves once the individual is able to adapt to the situation (Ullrich & Fitzgerald 2013:1014).

Student nurses were very overwhelmed when caring for intellectually disabled people. They felt depressed, scared, anxious, sad and heartbroken. They lost their drive and motivation to cope with the situation. This is consistent with a recent study by Janse Van Rensburg (2009:92) in which she claims that situations that contradict one’s values make it more difficult to find meaning within the context. She also states that value confrontations were linked to reflections on why intellectually disabled people were there and what their purpose was. Different emotions were stirred in these student nurses.

Student nurses were questioning the meaning of the lives of intellectually disabled people, and could not make sense of it. They asked themselves why the intellectually disabled are here in the world. Seachris (2013:33–35) reported that people frequently use the word ‘why’ to express a kind of cosmic complaint or bewilderment. The answer to the question ‘why are they here or exist’ is unknowable. For something to be unknowable, in principle it is impossible to know it.

The student nurses’ ability to think and reason was affected. Their own view on parenthood was influenced and they realised the needs of the intellectually disabled people. Caring for intellectually disabled people created doubts for student nurses regarding parenthood and whether they should or should not become parents. Being an effective parent is one of the most rewarding tasks in life and also one of the most challenging. Becoming a parent is an overwhelming responsibility of raising another human being. Effective parenting requires patience no matter the circumstances (Dinkmeyer & McKay 2000:1).

There were also compounding factors that affected student nurses while caring for intellectually disabled people. Intellectually disabled people are challenged in many ways due to underdeveloped processes of their brain. They react differently towards internal and external environments and their language and speech are affected. These noises had a negative effect on student nurses. Unusual sounds make a person more prone to feeling on edge and cause a loss of concentration. Lombard (2007:17) states that auditory overload is very real and a known occurrence. It depends on an individual as to how much noise she/he can cope with.

Due to so many intellectually disabled people being institutionalised, public psychiatric institutions are overcrowded. Human and material shortages make the situation to be more challenging. The wards appear to be unclean and unhygienic, with some rooms and surrounding areas foul-smelling and covered with faeces and urine. Student nurses were negatively affected by the smells that they encountered when caring for intellectually disabled people. The sense of smell affects the way an individual interact with others. When the sense of smell is bombarded with unpleasant input, an individual responds negatively to others within the environment and where there are more people, there is more sensory input. The more sensory input, the more sensory overload, leading to an individual getting stressed (Lombard 2007:21, 81).

Student nurses reported that seeing intellectually disabled people with severe physical deformities was traumatic and overwhelming. Janse Van Rensburg (2009:84) states that the initial experience of caring for intellectually disabled people could be described in terms of a confrontation with the unknown. The confrontation challenged the student nurses by creating intense emotional discomfort. Wong *et al*. (2004:201) further claim that any new experience, new encounter or uncertainty can evoke anxiety and people develop defence mechanisms to minimise feelings, thoughts and situations that they perceive as dangerous or uncomfortable. Student nurses experienced varying levels of discomfort and anxiety when caring for intellectually disabled people who are different from them in terms of physical appearance.

Student nurses experienced the way food was served to intellectually disabled people was inhumane. Intellectually disabled people require total nursing care and actual assistance in meeting all their needs in their activities of daily living in a long-term facility. According to Held (2006:32), ‘caring for’ entails meeting the needs of one person by another person, where the need is of such a nature that it cannot possibly be met by the person in need themselves.

Student nurses experienced intense emotional discomfort with regard to physical handling of intellectually disabled people. The discomfort was attributed to perceived lack of skill in handling these people. Feelings of discomfort include feeling overwhelmed and can lead to a need to escape. The discomfort facilitates student nurses to engage in a deeper emotional level with intellectually disabled people or to remain distant and aloof (Janse Van Rensburg 2009:215).

Student nurses were of the opinion that intellectually disabled people were treated unfairly with regard to poor quality nursing care, being abused and discriminated against. O’Donoghue (2004:81) states that nursing care of intellectually disabled people is goal-oriented to provide quality care within a caring environment, as opposed to a curing environment. Within this environment, nursing personnel should be educated to provide physical, ethical, moral and spiritual care. Furthermore, the Constitution of the Republic of South Africa (1996:7) states that no person may unfairly discriminate directly or indirectly against anyone. Held (2006:15) agrees that the ethics of justice focuses on the question of fairness and equality. All people are of equal value, deserving of basic human rights and dignity. Student nurses experienced an awareness that families and communities need to take responsibility to care for intellectually disabled people and need empowerment to become involved. Bauer and Shea (2003:59) agree that families can and do positively adapt to having a member with disability. After adaptation, families develop and find external support from work, churches and the community at large.

Student nurses experienced a sense of compassion and a new way of looking at life. They developed awareness for caring for intellectually disabled people. The intellectually disabled people, irrespective of their mental and physical disabilities, were accepted as human beings by the student nurses. The findings are consistent with a recent study conducted by Janse Van Rensburg (2009:99), in which a student nurse voiced an attitude of empathy by saying, ‘I realised as I went along, they are people like us. They are mentally challenged but at the other hand they are just people like us and they need us to understand who they are and where they belong’.

During the period of clinical placements, student nurses realised that intellectually disabled people also have needs. They need social contact. The most prevalent problem among intellectually disabled people is the sense of social isolation and social skills deficits. Improving the quantity and quality of social competence is a critical part of their care. More human and material resources are needed to provide quality care (Sadock & Sadock 2009:29).

Clinical practice is the core of nursing education during which student nurses are socialised into the nursing profession. Nash *et al*. (2010:669) state that to facilitate optimal learning for student nurses, they must be presented with a range of real-life work experiences that are presented in a supportive environment. Learning from experience gives one a holistic integrative perspective on learning that combines experience, perception, cognition and behaviour (Cross & Israelit 2000:313).

Student nurses learnt to appreciate their own level of health after seeing the conditions and deformities that the intellectually disabled people have. Gratitude has a tremendously positive value on helping people cope with daily problems, especially stress (Emmons 2013:10). Student nurses perceived caring for intellectually disabled people as stressful, but by comparing their lives with those of the intellectually disabled people, they appreciated what they had. They became grateful for the countless blessings in their lives.

Student nurses were able to embrace the opportunities of life. They appreciated the way in which the uniqueness of intellectually disabled people challenged and stretched their capacity for compassion and wisdom, acceptance and understanding. The gratitude for what a person gives extends to giving to those who are less fortunate (Emmons & McCullough 2004:173). Caring or feeling empathy for intellectually disabled people causes sympathy, compassion and unselfish tendencies to help.

Student nurses experienced a need to be supported while caring for intellectually disabled people in a public psychiatric institution. Student nurses reported that the clinical environment was overwhelming and tutors were not available to provide support. This finding is supported by Emanuel and Pryce-Miller (2013:19) who reported that particular needs of student nurses in the clinical environment are not met. Student nurses regarded tutors as a source of support and guidance; however, they were left to rely on ward sisters who also could not offer guidance, clinical teaching and supervision necessary due to a heavy workload and shortage of staff.

Student nurses experienced that they lack knowledge and skill to physically handle the intellectually disabled people. They were aware of their shortcomings but did not have a platform to express their feelings because of inadequate accompaniment. This is consistent with Mabuda’s (2008:24) findings that it is a challenge for student nurses to apply theory to practice, mainly because the theoretical content of the curriculum is too idealistic and academic and bears little relationship to the real needs of clinical practice. Student nurses will not know how to render effective client care in clinical settings unless they are shown how to correlate the theory they have been taught in training with real work situations.

Student nurses reported that it was challenging to care for intellectually disabled people and nursing personnel were not helpful in giving them guidance and support. The institution’s personnel were not good role models.

A reluctance to act as role models and mentors was observed from the institution’s personnel; they were always busy. Student nurses learn more effectively in an environment that facilitates learning by encouraging and supporting, and making them feel that they are part of the team (Emanuel & Pryce-Miller 2013:19).

Student nurses are working in a rapidly changing and stressful environment. They need more emotional support to develop compassionate care. Student nurses indicated that they need to be emotionally prepared before working in a public psychiatric institution.

Gaberson and Oermann (2010:50) reported in their study that most student nurses have anxiety about clinical learning activities and the environment, which hinders concentration and learning. They also said that orientation to the clinical facility before or on the first day of the clinical placement and activities is very important. It was evident from the interviews that student nurses lacked support. It was also apparent that they need various types of support systems in their clinical setting. Group interaction provides personal learning by way of other people through sharing information, gaining direct feedback or by direct suggestions (Ender & Newton 2000:132). On an emotional level, people gain qualities of support from the group, such as a sense of belonging, respect, caring and hope (Bergh & Theron 2012:193).

Structured moments for reflection could be helpful in the prevention of disproportional stress levels, low morals and burnouts experienced by students. Becker (2009:96) states that a student will take a single learning experience and reflect upon it so that he or she can make it effective by applying the experience to another challenge.

Student nurses were perplexed and confounded as to why God allowed the suffering and meaningless lives of these people. They experienced a need for guidance by a spiritual director. Spirituality is important for student nurses because they need to be spiritually centred and as clear as possible before they can be available to others. When meaninglessness and futility threaten to overwhelm order and rationality, student nurses as human beings in extreme circumstances need forms of solace (Webster 2002:112–113).

## Limitations of the study

It was challenging for the researcher to recruit student nurses; some refused to be interviewed and offered to write naïve sketches instead.

## Recommendations

Recommendations are made for psychiatric nursing education, practice and research.

### Psychiatric nursing education

Clinical nurse tutors should accompany student nurses in their clinical placements, to facilitate learning. Preceptorship and supervision of student nurses should encourage and promote learning, and facilitate their mental health.

### Psychiatric nursing practice

It is evident that the student nurses were overwhelmed by caring for intellectually disabled people in a public psychiatric institution. There is a need for student nurses to be supported when in clinical placements to help them cope. According to the Theory for Health Promotion in Nursing (University of Johannesburg 2010:4), student nurses’ mental health must be facilitated holistically; body, mind and spirit in interaction with the environment.

### Psychiatric nursing research

To improve psychiatric care and practice, research should be encouraged to promote evidence-based nursing. The researcher would recommend that this study be done again in different contexts involving a larger population of student nurses from different colleges placed in different psychiatric institutions for intellectually disabled people. This will improve the quality of care of intellectually disabled people and create a conducive learning environment for student nurses.

## Conclusion

The findings indicate that student nurses experience challenges that can have an impact on their body, mind and spirit when caring for intellectually disabled people. Coping strategies ranged from orientation in clinical placements, accompaniment debriefing before and after clinical placements, constructive interpersonal relationships in clinical areas and student nurses taking care of themselves through self-awareness and by using peer support.

It can thus be concluded that the purpose of the study was reached because the findings provide a better understanding of the lived experiences of student nurses caring for intellectually disabled people in a public psychiatric institution.
